# Secular Trends in Dietary Intake over a 20-Year Period in People with Type 2 Diabetes in Japan: A Comparative Study of Two Nationwide Registries; Japan Diabetes Complications Study (JDCS) and Japan Diabetes Clinical Data Management Study (JDDM)

**DOI:** 10.3390/nu13103428

**Published:** 2021-09-28

**Authors:** Mizuki Takeuchi, Chika Horikawa, Mariko Hatta, Yasunaga Takeda, Rina Nedachi, Izumi Ikeda, Sakiko Morikawa, Noriko Kato, Hiroki Yokoyama, Rei Aida, Shiro Tanaka, Chiemi Kamada, Yukio Yoshimura, Toshiko Saito, Kazuya Fujihara, Atsushi Araki, Hirohito Sone

**Affiliations:** 1 Department of Hematology, Endocrinology and Metabolism, Faculty of Medicine, Niigata University, Niigata 951-8510, Japan; y.mizuki3929@gmail.com (M.T.); mr2.yac@gmail.com (Y.T.); rina.nedachi.t@gmail.com (R.N.); ikdizm12@gmail.com (I.I.); kafujihara-dm@umin.ac.jp (K.F.); 2 Department of Health and Nutrition, Niigata University of Health and Welfare, Niigata 950-3198, Japan; saito@nuhw.ac.jp; 3 Department of Health and Nutrition, Faculty of Human Life Studies, University of Niigata Prefecture, Niigata 950-8680, Japan; horikawa@unii.ac.jp; 4 Saiseikai Niigata Hospital, Niigata 950-1104, Japan; marichocolate.coffee02@gmail.com; 5 Department of Food Science and Dietetics, Faculty of Human Life Studies, Tokushima Bunri University, Tokushima 770-8514, Japan; sakiko.1211@gmail.com; 6 Kato Clinic of Internal Medicine, Tokyo 125-0054, Japan; norikokato.0131@gmail.com; 7 Jiyugaoka Medical Clinic, Obihiro 080-0016, Japan; dryokoyama@yokoyamanaika.com; 8 School of Medicine, Osaka City University, Osaka 545-8585, Japan; aida.rei@med.osaka-cu.ac.jp; 9 Department of Clinical Biostatistics, Graduate School of Medicine, Kyoto University, Kyoto 606-8501, Japan; tanaka.shiro.8n@kyoto-u.ac.jp; 10 Faculty of Human Life Science, Shikoku University, Tokushima 771-1192, Japan; c-kamada@shikoku-u.ac.jp (C.K.); yyoshimura@shikoku-u.ac.jp (Y.Y.); 11 Department of Diabetes, Metabolism and Endocrinology, Tokyo Metropolitan Geriatric Hospital, Tokyo 173-0015, Japan; tsu-ara140@nifty.com

**Keywords:** food intake, type 2 diabetes, obesity, diabetes mellitus, Asia

## Abstract

Background: In order to provide effective dietary guidance, it is necessary to consider dietary intake, which can change over time. This study analyzed changes in the diet of Japanese patients with type 2 diabetes over a 20-year period. Methods: We compared the results of two dietary surveys that used the food frequency questionnaire format. The first was conducted in 1996 by the Japan Diabetes Complications Study (JDCS) (*n* = 1509; males 53.3%), and the second in 2014–2018 by the Japan Diabetes Clinical Data Management Study (JDDM) (*n* = 1145; males 65.6%). Both are nationwide representative registries of outpatients with type 2 diabetes in Japan. Results: Over a 20-year period, both men and women with type 2 diabetes had a significant increase in body mass index (BMI). Nonetheless, there was only a small change in energy intake. Conversely, there was a significant increase in fat intake and thus in the fat-to-energy ratio. With regard to food groups, there was a significant increase in meat intake and a decrease in the intake of fish, soybeans/soy products, vegetables, and fruits, with a particularly significant decrease in vegetables. Conclusions: Even in Japan, an industrialized country with a stable socioeconomic environment, there were many significant changes in the dietary intake of patients with type 2 diabetes over the 20-year period.

## 1. Introduction

Diet plays a central role in diabetes self-management education and support [1]. To provide effective dietary guidance and/or to establish contemporary dietary guidelines, we must understand and take into account the changes in dietary intake and pattern over time. Dietary habits, including dietary content, are strongly influenced by changes in socioeconomic conditions and the environment [2]. The National Health and Nutrition Survey in Japan revealed decreases in intakes of 151 kcal (−7.4%) of energy, 24.9 g (−8.9%) of carbohydrates, 12.7 g (−15.5%) of protein, and 3.8 g (−27.7%) of salt (as sodium chloride) over a period of 20 years (1996–2016).

People with diabetes often receive nutritional guidance in the early stages of the disease, so their knowledge, as well as dietary content and habits, may differ from those who do not have diabetes. Thus, dietary changes in people with type 2 diabetes may differ from the general population. However, very few studies have investigated this change. In a study reporting on the dietary changes made by people with type 2 diabetes in the United States over the past 25 years, Casagrande et al. [3] reported 206 kcal (+12.2%) and 339 mg (+11.2%) increases in energy and sodium, respectively, as well as a 0.9 g (−8.7%) decrease in dietary fiber. In addition, according to the Dietary Survey of Type 2 Diabetes in Spain [4], although the sample size was small at 200, protein intake decreased by 6.9% in men and 1.4% in women, and fat intake increased by 3.2% in men and 5.5% in women in 2000 compared to 1993. However, as these studies focused on nutrient changes, it was unclear what changes in foods contributed to these nutrient changes. Dietary intake by food groups in those with type 2 diabetes has been previously reported [5], but to the best of our knowledge, there have been no studies on changes in dietary intake over time.

The diabetic population in the Asian region is rapidly increasing, with those in East Asia accounting for approximately a quarter of the world diabetes population [6]. Therefore, from the perspective of global epidemiology, it is important to elucidate changes in the nutrient intake of Asian people with diabetes over time. Previously, we reported [7] on the nutritional status of Japanese patients with type 2 diabetes from a survey conducted in 1996. However, no study thus far has examined changes in nutrient intake in the Asian diabetic population over time on a nationwide scale.

The purpose of this study was to reveal the detailed changes in dietary intake in Japanese patients with type 2 diabetes over a 20-year period beginning in 1996. In the present study, we conducted a nutritional survey, almost identical to our previous survey, which focused on a newly established nationwide registry of Japanese patients with type 2 diabetes. We then compared the results with those of our previous study. The results of this study reveal the importance of understanding which areas need to be focused on for efficient and effective dietary guidance, and thus for the creation of contemporary dietary guidelines. In addition, the results may provide fundamental insights into the differences in dietary advice received by patients of Asian and Western origins.

## 2. Materials and Methods

This study compared the results of two dietary surveys and the associated clinical variables regarding participants in nationwide registries of outpatients with type 2 diabetes in Japan, namely the Japan Diabetes Complications Study (JDCS) [8] and the Japan Diabetes Clinical Data Management Study (JDDM). For the dietary surveys, both used an almost identical format to the food frequency questionnaire based on food groups (FFQg) [9]. The JDCS is a nationwide cohort study of patients with type 2 diabetes aged 40–70 years, with HbA1c levels of 6.5% or higher, and who attended outpatient clinics at 59 hospitals [8]. Of the 2033 enrollees, 1509 who responded to the self-administered dietary survey in 1996 were included in the analysis. The JDDM is another nationwide registry of outpatients with type 2 diabetes, but which started later than the JDCS. The JDDM conducted its dietary survey in 2014–2018 with essentially the same format as that used by the JDCS. We used the survey results from 1145 patients aged 40–70 years [10].

The nutrient and food intakes in both studies were assessed using the FFQg (JDCS: FFQg 1st edition, JDDM: FFQg v3.5). FFQg is a questionnaire that solicits information on the intake of 29 food groups and 10 cooking methods. An evaluation can be made on the average weekly intake for each food or food group in commonly used units or portion sizes. The validity of the FFQ was shown by comparison with consecutive 7-day weighed dietary records of 66 individuals aged 19–60 years [11]. We calculated the nutrient and food group intakes using standardized nutrition software (EIYO-KUN (JDCS: v4.5, JDDM: v6.0)) that is used in surveys and nutrition counseling in Japan. The FFQ v3.5 used in the JDDM calculates grains and seaweed in a rehydrated condition, while the JDCS calculates these products as a dry food. Therefore, in the present analysis, the values from the JDDM survey were converted to dry matter values for comparison of food conditions with the JDCS survey. The differences between EIYO-KUN v4.5 and v6.0 consist of additional listings of food composition, so the method for the calculation of nutrients was not changed.

In addition to dietary surveys, information from physical examinations and blood tests was also evaluated. Body mass index (BMI) was calculated as weight (kg) divided by the square of height (m). Blood tests were carried out at each clinic. All data are presented as mean ± standard deviation or percentage. The comparisons of participants’ characteristics, nutrient intakes, and intakes by food group were examined by the unpaired Student’s t-test, chi-square (χ^2^) tests, and an analysis of covariance. In the analysis of covariance, each of the nutrients was used as a dependent variable, and age, HBA1c, BMI, treated by insulin (yes or no), and treated by OHA (yes or no) were used as adjustment variables. All statistical analyses were carried out using SPSS software (v26.0) and the significance level was 0.05.

This study was in accordance with the Declaration of Helsinki and the ethical guidelines for clinical-epidemiological studies by the Japanese Ministry of Health, Labour and Welfare; the JDCS was approved by the review boards of all participating institutions; and the JDDM was approved by the ethical review boards of the JDDM and Niigata University (2015–1648). Informed consent was obtained from all individual participants included in the study.

## 3. Results

Table 1 shows the clinical features of participants according to registry and sex. Although statistically significant differences were found between JDCS and JDDM patients in most variables, the differences in age and diabetes duration were too small to be considered clinically different. Variables that were considered to be clinically different between registries included BMI, HbA1c, blood pressure, and smoking rate among men. The BMI was significantly higher (+3.1 kg/m^2^ in men and +2.7 kg/m^2^ in women) in JDDM participants compared to JDCS participants. Comparing the mean BMI between JDCS and JDDM participants, the proportion of obese patients defined by BMI ≥ 25 kg/m^2^ according to Japanese criterion was significantly greater in JDDM participants, being approximately 20% to 50% higher in men and approximately 30% to 50% higher in women. HbA1c was significantly lower by approximately 1% and systolic blood pressure by approximately 5 mmHg for both men and women among JDDM participants, and the difference in the smoking rate for men was approximately 15% lower in JDDM participants than in JDCS participants.

When participants were divided into two groups based on the median age of 60 years, the characteristics were similar to those identified in the comparison of all groups, regardless of age. However, there was a dramatic increase in the BMI of JDDM participants younger than 60 years compared to JDCS participants in the same age group (Supplementary Table S1).

Table 2 shows a comparison of daily nutrient intake between patients in both studies. In Model 2, which was adjusted for age, HbA1c, BMI, insulin use, and oral medication use, there was no significant difference in energy intake in men between the two studies, but in women, there was a slight, but significant, increase of approximately 5% in the JDDM compared to the JDCS. Regarding proportions of macronutrients, the fat-to-energy ratio increased significantly, by about 3%, and the carbohydrate-to-energy ratio and protein-to-energy ratio decreased significantly for both men and women from the JDCS to the JDDM. Regarding changes in fat intake, the proportion of energy derived from saturated fatty acids increased significantly from 7.6% to 9.0% in men and from 8.3% to 9.4% in women in the interval between the surveys, whereas the proportion of energy derived from polyunsaturated fatty acids decreased significantly. Other variables that showed nutritionally meaningful differences between the two studies included significant decreases in dietary fiber, by 1.7 g for men and 1.6 g for women, and in salt, by 1.9 g for men and 2.7 g for women. The results were similar to the comparison of all groups, with fat-to-energy ratio and saturated fatty acid-to-energy ratio significantly increased in JDDM patients regardless of age, and polyunsaturated fatty acid-to-energy ratio, dietary fiber, and salt significantly decreased in JDDM patients (Supplementary Table S2).

Table 3 shows a comparison of daily food intakes categorized by food groups. In terms of grains intake, from the JDCS to the JDDM, there was a slight, but significant, decrease of 7.4% in men, but no significant difference was found in women. Meat intake by JDDM patients was significantly increased, by about 60%, for both men and women compared to JDCS patients, and intake of fish and soybean/soy products was significantly decreased in JDDM patients. In particular, changes in meat and fish intake were remarkable, with an increase of about 30 g of meat in the JDDM participants and a decrease of about 30 g of fish in the JDDM participants, for both men and women. In summary, the JDCS patients consumed more fish and soybean/soy products than meat, while the JDDM patients consumed more meat than fish and soybean/soy products; thus, the composition of the major protein food sources was reversed over the 20-year period. In addition, dairy intake decreased by about 20%, and intake of sweets and snacks was about three times higher in that interval. On the other hand, the intake of vegetables and fruits among JDDM patients was dramatically reduced by about 30% in both men and women compared with JDCS patients. Both intakes of green-yellow vegetables and other vegetables significantly decreased from the time of the JDCS survey to the JDDM survey. In particular, intake of green-yellow vegetables was markedly reduced between the two surveys by 42.0% for men and 35.0% for women. Regardless of age, meat intake increased in JDDM patients, while fish and soybean/soy products showed a decrease. However, there was no trend toward a reversal of the composition of the major protein food sources in patients aged 60 years and older. In both the under 60 and over 60 age groups, there was an increase in sweets and snacks and a decrease in fruits and total vegetable intake (Supplementary Table S3).

## 4. Discussion

This study analyzed changes in the diet of Japanese patients with type 2 diabetes over a 20-year period using two surveys with essentially the same format. Although the backgrounds of the patients, including age and duration of diabetes, were quite similar between the two studies, BMI and obesity rates were markedly increased in the later study. Regarding dietary content, fat intake increased, including saturated fatty acids, along with significant increases in the consumption of meat and sweets and snacks, and a significant decrease in vegetable and fish intake. To date, there have been no studies on the changes in dietary intake by persons with type 2 diabetes in Asia, except for a report on adherence to dietary recommendations in Korea [12]. To our knowledge, the changes in nutrient and food intake have not been reported. Therefore, this is the first study to clarify the secular changes in the dietary status of patients with type 2 diabetes in Asia.

Self-management education and support for diet and physical activity are essential in the care of overweight or obese people with type 2 diabetes. A previous study observing the secular trends in the United States from 1988 to 2012 [3] showed a trend of increases in BMI and energy intake in patients with type 2 diabetes, which was also the case even in adults without diabetes. On the other hand, in our study of East Asians, the BMI markedly increased from 22.7 kg/m^2^ to 25.8 kg/m^2^ in men and 23.2 kg/m^2^ to 25.9 kg/m^2^ in women, although no such remarkable changes were found in the Japanese general population according to the Japan National Health and Nutrition Survey performed during a similar period (1996 and 2016) (from 23.3 kg/ m^2^ to 24.0 kg/m^2^ in men and 23.2 kg m^2^ to 22.6 kg/m^2^ in women) [13]. This suggests that secular changes in BMI and obesity were different between people with and without type 2 diabetes and between the East and the West. According to the National Health and Nutrition Surveys, from 1996 to 2016, the energy intake by the general population aged 40–69 years mildly declined from 2284 kcal to 2145 kcal (−139 kcal) for men and from 1880 kcal to 1722 kcal (−158 kcal) for women, although the dietary survey method was different from our method [14].

We previously reported, using JDCS baseline data, that Western populations with diabetes are mostly obese and are also more obese than the general population, whereas Japanese people with diabetes not only have a mean BMI within the normal range, but also it is almost the same as in the general population [15]. The results of the current study, including the JDDM results 20 years after the initial JDCS survey, strongly support the fact that obesity among those with type 2 diabetes has recently increased in Japan, as is the case in Western countries [16]. On the other hand, it is not clear from this study alone why the increase in BMI in Japanese patients with type 2 diabetes was significantly greater than in the general population, even though there was no significant increase in energy intake between the two studies. Our previous study [17] suggested that there are racial differences in the relationship between energy intake and obesity, but several other hypotheses are also possible.

The first possible explanation for the significant increase in BMI without a significant increase in energy intake in Japanese patients with type 2 diabetes may be the effect of decreased energy expenditure due to decreased physical activity. A comparison of the activity levels between people with diabetes and the general population found no significant difference in physical activity levels and leisure-time physical activity patterns, and reported that neither groups achieved the recommended physical activity goals [18,19]. The decrease in the number of steps by the general Japanese population from 1996 to 2016 was remarkable for both men and women [13], and it was pointed out that the decrease in physical activity was due to the development of transportation [20]. In this study, direct comparisons were difficult because of the differences in the studies’ survey methods regarding the amount of physical activity; however, 44.4% of patients in the JDDM reported low levels of overall physical activity in their daily lives, which was calculated from activity time and activity intensity. Future research on secular trends in the amount of physical activity in patients with type 2 diabetes is expected.

The second theory as to why BMI increased without an increase in energy intake is that it could be due to the effect of smoking rates and medications. The BMI of smokers was reportedly lower than that of non-smokers [21,22], and the smoking rate of men in the JDDM with a high BMI was significantly lower than that in the JDCS. In addition, the increase in the rate of insulin use over time that was observed between these studies may be a factor in weight gain. The fact that there was a significant decrease in the rate of use of oral hypoglycemic agents may have contributed to weight gain or weight loss, depending on the type of oral hypoglycemic agent [23]. Further studies, including a detailed follow-up of the status of the medications, are necessary.

As a third conjecture, the ratio of macronutrients may have affected these findings. Regarding the energy-producing nutrients, both men and women in the JDDM had higher fat-to-energy ratios than those in the JDCS. In the JDDM compared to the JDCS, men had a lower carbohydrate-to-energy ratio and protein-to-energy ratio and women had a lower protein-to-energy ratio. Women in the JDDM had an increase in energy intake, which could be attributed to an increased fat intake as their carbohydrate and protein intakes did not increase. In the Japanese National Health and Nutrition Survey, which was of a similar duration (1996 and 2016), the fat-to-energy ratio increased (men: +2.6%, women: +2.9%), but carbohydrate and protein intakes tended to decrease (carbohydrate: −22.6 g/day for men, −33.4 g/day for women, protein: −15.9 g/day for men, −13.0 g/day for women), and fat intake was almost unchanged, suggesting that the fat-to-energy ratio was relatively increased. Increased fat-to-energy ratios due to an increased fat intake in patients with type 2 diabetes may have contributed to the increased BMI. However, previous studies have not provided a unified consensus on the association between the fat-to-energy ratio and obesity, which needs to be clarified in the future [24].

It is well known that, regardless of body weight, a high-fat diet, especially an excessive intake of saturated fatty acids and a high saturated fatty acid-to-energy ratio, is significantly associated with a higher risk of cardiovascular events in patients with type 2 diabetes [25]. According to the Japanese practice guidelines for diabetes [26], a reduction in saturated fatty acids is recommended when the lipid-to-energy ratio exceeds 25%. In addition, *Dietary Reference Intakes for Japanese* [27] set a target saturated fatty acid-to-energy ratio of ≤7%. Among our patients with a fat-to-energy ratio of ≥25%, only 9.5% of JDCS patients and 4.4% of JDDM patients had a saturated fatty acid-to-energy ratio of ≤7%, suggesting the necessity for enhanced guidance to reduce the saturated fatty acid-to-energy ratio. The saturated fatty acid-to-energy ratio for Americans with type 2 diabetes was approximately 11%, which was still higher than the level (<10%) recommended by the U.S. Dietary Guidelines, but it did not increase between 1988 and 2012 [28].

In contrast, in our Japanese patients, the saturated fatty acid-to-energy ratio increased significantly over the 20 years to approximately 9% in both men and women, and the percentage of patients with a saturated fatty acid-to-energy ratio of more than 10% increased significantly from 8.2% in men and 13.3% in women in the JDCS to 28.4% in men and 36.6% in women in the JDDM. This suggests that the fat intake pattern of Japanese people with type 2 diabetes is approaching that of Western patients. Therefore, correcting excessive lipid intake while taking into account the content and quality of lipids is considered to be a crucial issue.

In terms of secular changes in food group intake patterns, an increase in meat intake and a decrease in the consumption of fish and dairy products were observed as sources of protein intake. JDCS patients had higher intakes of fish and soybeans/soy products than meat, whereas JDDM patients had higher intakes of meat than fish and soybeans/soy products. The National Health and Nutrition Survey [29] also showed the same secular changes in food group intake patterns as this study.

It is indicated that patients with type 2 diabetes in Japan had a major change in the composition of their protein sources, as did the general Japanese population, which was a change to a meat-centered diet. In general, among people in Western countries, the circumstances in which meat intake is higher than fish intake continues. It is inferred that the diet of Japanese patients with type 2 diabetes has been transformed into a diet centered on meat due to the influx of various lifestyle patterns and dietary cultures from Western countries, as is the case with the general Japanese population. It has been reported that meat was associated with cardiovascular complications and mortality in people with type 2 diabetes in a study in the US [30] and in our previous study [31]. Thus, it is considered necessary to strengthen the guidance on protein sources to prevent complications in the future.

Meat is the food with the highest contribution to fat intake by Japanese people. Therefore, it is thought that an increased meat intake may also affect fat-to-energy ratios and saturated fatty acid increases in JDDM patients. Aside from meat, saturated fatty acids are found in the Japanese diet in milk, butter, and coconut oil, which are commonly used as ingredients in Western sweets rather than in traditional Japanese ones (which are mostly made with sugar, rice, and beans [32]). Although the type of sweets could not be identified in this survey, as their intake more than doubled from the JDCS to the JDDM, it is inferred that in conjunction with meat, the increase in the intake of sweets contributed to the increase in saturated fatty acid consumption.

Contrary to meat, the intake of fruits and vegetables decreased significantly between the two studies. As vegetables and fruits are good sources of dietary fiber, fiber intake decreased significantly as vegetable and fruit intakes decreased. A higher intake of dietary fiber is known to improve glycemic control, and we previously reported that it was significantly associated with a reduced risk of stroke [33] and retinopathy [34] in JDCS patients. However, despite its importance, the intake of dietary fiber was lower than the recommended goal of 20 g/day or higher in 1996, and even declined in the following 20 years. A decreased intake of fruits and vegetables is apparent in the Japanese general population [29], and is considered to be an important issue in dietary guidance.

As to the background of the decrease in the consumption of vegetables and fruits, survey reports have suggested that differences in lifestyle, such as economic conditions [35] and living alone, exist [36] and that diabetic patients who work may choose a less diversified diet [37]. It was also reported that the consumption of vegetables and fruits tends to be higher with a lower frequency of eating meals away from home [38]. As to the proportions of food expenditure in Japan from 1995 to 2015, the consumption of fresh foods has decreased, and eating out and consuming processed foods have increased from 65.4% to 72.6% [39]. This suggests that the increase in eating out and buying processed foods may influence the decrease in fruit and vegetable intake, not only in the general population, but also in those with type 2 diabetes. This Westernization of dietary habits has been widely adopted, and advice on the dietary treatment of diabetes should consider the current dietary situation.

This study has several limitations. First, both the JDCS and the JDDM were comprised of patients from different cohorts, although they were both multicenter studies. However, both studies were conducted at outpatient clinics throughout Japan specializing in diabetes, and the clinical settings were similar except for the time period studied. An additional limitation is that the number of participants that were included in both surveys was different from the potential candidates. Of the 2033 JDCS patients, 1588 responded to a baseline dietary survey, and 524 patients who had missing values in the dietary survey were excluded. In the JDDM patients, 1145 of 2337 patients met the eligibility criteria for the JDCS and responded to the baseline dietary survey. However, there were no notable differences in baseline characteristics between the responders and non-responders [8], and consequently, no selection bias was found. Finally, the participants’ dietary intake was obtained from a self-administered questionnaire, which could be an underestimation. As for the long-term comparison of 20 years, changes had been made in the food composition tables and in the FFQg version used in both studies. However, those changes corresponded to changes in dietary habits and food ingredients due to secular trends, and there was little effect on energy intake and intake of macronutrients due to the changes in the food composition tables.

The results of this study clarify the changes in the dietary intake of Japanese patients with type 2 diabetes over 20 years, beginning in 1996. Despite a lack of significant change in the total energy intake during the period, both men and women with type 2 diabetes had a large increase in BMI. A rise in the fat-to-energy ratio due to an increase in fat intake was also observed, particularly in men. In terms of intake by food group, both men and women had clearly unfavorable changes in terms of complication prevention. These included an increase in the intake of meat and a decrease in the intake of fish, soybean/soy products, vegetables, and fruits. The results of our study strongly suggest that, in order to prevent complications in Japanese patients with type 2 diabetes in the future, the provision of nutritional guidance, in line with these chronological changes in dietary intake, is necessary.

## Figures and Tables

**Table 1:** Clinical characteristics of participants in two studies of Japanese type 2 diabetes patients 20 years apart, i.e., the Japan Diabetes Complications Study (JDCS, 1996) and the Japan Diabetes Clinical Data Management Study (JDDM, 2014–2018).

	Men			Women		
	JDCS (1996)	JDDM (2014–2018)	*p* Value	JDCS (1996)	JDDM (2014–2018)	*p* Value
	(*n* = 804)	(*n* = 751)		(*n* = 705)	(*n* = 394)	
Age (y)	58.4 ± 7.0	57.6 ± 8.6	0.048	59.0 ± 6.8	60.1 ± 7.7	0.011
Height (cm)	165.2 ± 6.0	168.9 ± 6.3	<0.001	152.7 ± 5.0	155.2 ± 5.5	<0.001
Weight (kg)	62.0 ± 8.6	73.8 ± 14.0	<0.001	54.2 ± 8.3	62.5 ± 13.4	<0.001
BMI (kg/m^2^)	22.7 ± 2.6	25.8 ± 4.2	<0.001	23.2 ± 3.3	25.9 ± 5.2	<0.001
BMI≧25 kg/m^2^ (%)	19.3	53.5	<0.001	28.2	52.8	<0.001
Diabetes duration (years)	11.5 ± 7.4	10.7 ± 7.0	0.028	10.4 ± 6.7	10.6 ± 7.3	0.579
HbA1c (%) (mmol/mol)	8.2 (66) ± 1.3	7.2 (55) ± 1.2	<0.001	8.5 (69) ± 1.4	7.3 (56) ± 1.0	<0.001
Systolic blood pressure (mmHg)	131.2 ± 15.7	126.8 ± 14.4	<0.001	131.7 ± 16.3	126.1 ± 15.2	<0.001
Diastolic blood pressure (mmHg)	77.2 ± 9.8	73.5 ± 11.3	<0.001	76.0 ± 9.9	70.8 ± 9.6	<0.001
Total serum cholesterol (mg/dL)	193.6 ± 34.9	190.0 ± 32.8	0.056	209.4 ± 33.5	201.0 ± 33.5	<0.001
Serum HDL-C (mg/dL)	52.4 ± 16.6	53.2 ± 15.4	0.374	56.8 ± 16.9	61.7 ± 14.7	<0.001
Serum non-HDL-C (mg/dL)	141.2 ± 36.3	136.7 ± 35.5	0.030	152.2 ± 34.2	137.8 ± 32.2	<0.001
Treated by insulin (%)	18.2	19.7	0.009	22.0	24.4	0.010
Treated by OHA without insulin (%)	60.2	55.0	<0.001	61.7	50.3	<0.001
Current smoker (%)	43.8	28.9	<0.001	8.2	8.9	0.882

Abbreviations: HbA1c, hemoglobin A1c; HDL-C, high-density lipoprotein cholesterol; OHA, oral hypoglycemic agents. Data are mean ± SD.

**Table 2:** Comparison of each nutrient intake in male and female participants of JDCS (1996) and JDDM (2014–2018).

		Men						Women					
		Mean ± SD	*p* Value	Model 1		Model 2		Mean ± SD	*p* Value	Model 1		Model 2	
				Mean ± SEE	*p* Value	Mean ± SEE	*p* Value			Mean ± SEE	*p* Value	Mean ± SEE	*p* Value
Energy (kcal)													
	JDCS	1818 ± 399.9	0.200	1818 ± 16.3	0.285	1813 ± 17.0	0.399	1643 ± 406.0	0.025	1636 ± 15.6	0.010	1631 ± 16.1	0.016
	JDDM	1846 ± 454.5		1845 ± 17.0		1838 ± 19.8		1697 ± 377.6		1709 ± 21.8		1710 ± 25.4	
Carbohydrate													
%Energy	JDCS	58.1 ± 6.4	<0.001	58.2 ± 0.3	<0.001	58.2 ± 0.3	<0.001	55.1 ± 6.2	0.032	55.2 ± 0.2	0.024	55.2 ± 0.2	0.203
	JDDM	56.0 ± 6.9		56.0 ± 0.3		56.0 ± 0.3		54.3 ± 5.8		54.2 ± 0.3		54.6 ± 0.4	
g	JDCS	240 ± 55.5	0.093	239 ± 2.3	0.209	239 ± 2.3	0.221	220 ± 48.6	0.654	219 ± 1.9	0.290	219 ± 1.9	0.165
	JDDM	235 ± 63.4		235 ± 2.4		234 ± 2.7		221 ± 46.9		223 ± 2.7		224 ± 3.1	
Protein													
%Energy	JDCS	15.2 ± 2.3	<0.001	15.2 ± 0.1	<0.001	15.1 ± 0.1	0.001	16.2 ± 2.4	<0.001	16.2 ± 0.1	<0.001	16.2 ± 0.1	<0.001
	JDDM	14.6 ± 2.2		14.7 ± 0.1		14.6 ± 0.1		15.1 ± 2.1		15.1 ± 0.1		15.0 ± 0.2	
g	JDCS	69.6 ± 20.7	0.026	69.4 ± 0.8	0.117	69.2 ± 0.8	0.124	67.1 ± 22.7	0.028	67.0 ± 0.8	0.100	66.7 ± 0.9	0.139
	JDDM	67.3 ± 19.2		67.5 ± 0.8		67.1 ± 0.9		64.3 ± 16.7		64.5 ± 1.2		64.1 ± 1.4	
Fat													
%Energy	JDCS	26.7 ± 4.9	<0.001	26.7 ± 0.2	<0.001	26.6 ± 0.2	<0.001	28.7 ± 4.8	<0.001	28.6 ± 0.2	<0.001	28.6 ± 0.2	<0.001
	JDDM	29.4 ± 5.6		29.3 ± 0.2		29.4 ± 0.2		30.5 ± 4.9		30.7 ± 0.3		30.4 ± 0.3	
g	JDCS	54.3 ± 17.1	<0.001	54.4 ± 0.7	<0.001	54.1 ± 0.7	<0.001	53.2 ± 18.9	<0.001	52.9 ± 0.7	<0.001	52.7 ± 0.8	<0.001
	JDDM	60.7 ± 20.4		60.6 ± 0.7		60.4 ± 0.9		58.3 ± 18.5		58.9 ± 1.0		58.5 ± 1.2	
Saturated fatty acids (% Energy)													
	JDCS	7.6 ± 1.7	<0.001	7.6 ± 0.1	<0.001	7.6 ± 0.1	<0.001	8.3 ± 1.6	<0.001	8.3 ± 0.1	<0.001	8.3 ± 0.1	<0.001
	JDDM	9.0 ± 2.1		9.0 ± 0.1		9.0 ± 0.1		9.4 ± 2.0		9.5 ± 0.1		9.4 ± 0.1	
Monounsaturated fatty acids (% Energy)													
	JDCS	8.7 ± 2.0	<0.001	8.8 ± 0.1	<0.001	8.8 ± 0.1	<0.001	9.3 ± 2.0	<0.001	9.3 ± 0.1	<0.001	9.3 ± 0.1	<0.001
	JDDM	10.5 ± 2.5		10.4 ± 0.1		10.4 ± 0.1		10.5 ± 2.2		10.6 ± 0.1		10.5 ± 0.1	
Polyunsaturated fatty acids (% Energy)													
	JDCS	6.4 ± 1.5	<0.001	6.5 ± 0.1	<0.001	6.4 ± 0.1	<0.001	6.8 ± 1.5	<0.001	6.9 ± 0.1	<0.001	6.9 ± 0.1	<0.001
	JDDM	6.0 ± 3.4		5.9 ± 0.1		6.0 ± 0.1		6.3 ± 1.3		6.3 ± 0.1		6.2 ± 0.1	
n-6 Fatty acids (% Energy)													
	JDCS	5.2 ± 1.3	<0.001	5.2 ± 0.1	<0.001	5.2 ± 0.1	<0.001	5.5 ± 1.4	<0.001	5.5 ± 0.1	<0.001	5.5 ± 0.1	<0.001
	JDDM	4.8 ± 1.1		4.8 ± 0.1		4.9 ± 0.1		5.1 ± 1.1		5.1 ± 0.1		5.0 ± 0.1	
n-3 Fatty acids (% Energy)													
	JDCS	1.5 ± 0.4	<0.001	1.5 ± 0.01	<0.001	1.5 ± 0.01	<0.001	1.6 ± 0.4	<0.001	1.6 ± 0.02	<0.001	1.6 ± 0.02	<0.001
	JDDM	1.1 ± 0.3		1.1 ± 0.01		1.1 ± 0.02		1.2 ± 0.3		1.2 ± 0.02		1.2 ± 0.02	
Fiber (g)													
	JDCS	14.1 ± 5.3	<0.001	14.0 ± 0.2	<0.001	13.9 ± 0.2	<0.001	15.4 ± 5.3	<0.001	15.4 ± 0.2	<0.001	15.3 ± 0.2	<0.001
	JDDM	12.2 ± 3.9		12.3 ± 0.2		12.2 ± 0.2		13.7 ± 3.9		13.8 ± 0.3		13.7 ± 0.3	
Salt (g)													
	JDCS	10.5 ± 3.9	<0.001	10.5 ± 0.1	<0.001	10.4 ± 0.1	<0.001	10.9 ± 4.0	<0.001	10.9 ± 0.1	<0.001	10.9 ± 0.2	<0.001
	JDDM	8.4 ± 3.0		8.4 ± 0.1		8.5 ± 0.2		8.1 ± 2.5		8.1 ± 0.2		8.2 ± 0.2	

Values are mean ± SD obtained using *t*-test and mean ± SEE obtained using ANCOVA. Model 1 was adjusted for age, HbA1c (%), and BMI (kg/m^2^). Model 2 was adjusted as for Model 1, treated by insulin (yes or no), and treated by OHA (yes or no).

**Table 3:** Comparison of major food group intake among male and female participants in JDCS (1996) and JDDM (2014–2018).

	Men						Women					
	Mean ± SD	*p* Value	Model 1		Model 2		Mean ± SD	*p* Value	Model 1		Model 2	
			Mean ± SEE	*p* Value	Mean ± SEE	*p* Value			Mean ± SEE	*p* Value	Mean ± SEE	*p* Value
Grains (g)												
JDCS	207.5 ± 57.9	<0.001	206.3 ± 2.3	0.009	207.7 ± 2.4	<0.001	173.1 ± 40.0	0.595	171.8 ± 1.6	0.090	171.8 ± 1.6	0.096
JDDM	195.7 ± 63.1		196.9 ± 2.4		192.4 ± 2.8		174.4 ± 39.9		176.7 ± 2.2		177.2 ± 2.6	
Potato/aroid (g)												
JDCS	49.6 ± 40.2	<0.001	49.4 ± 1.3	<0.001	49.0 ± 1.3	<0.001	58.2 ± 49.9	<0.001	58.2 ± 1.7	<0.001	57.5 ± 1.8	<0.001
JDDM	22.8 ± 24.4		23.0 ± 1.3		24.0 ± 1.6		31.2 ± 27.4		31.2 ± 2.4		33.0 ± 2.8	
Vegetables total (g)												
JDCS	303.8 ± 171.0	<0.001	300.8 ± 5.5	<0.001	298.9 ± 5.8	<0.001	347.2 ± 163.2	<0.001	343.4 ± 5.9	<0.001	342.1 ± 6.1	<0.001
JDDM	219.0 ± 112.5		222.3 ± 5.7		217.7 ± 6.8		264.9 ± 116.6		271.8 ± 8.2		271.2 ± 9.6	
Green-yellow vegetables (g)												
JDCS	129.7 ± 68.7	<0.001	128.8 ± 2.2	<0.001	127.8 ± 2.3	<0.001	147.4 ± 65.6	<0.001	146.4 ± 2.4	<0.001	145.7 ± 2.4	<0.001
JDDM	74.0 ± 44.2		75.0 ± 2.3		74.1 ± 2.7		93.7 ± 47.0		95.7 ± 3.3		94.7 ± 3.9	
Other vegetables (g)												
JDCS	174.1 ± 103.4	<0.001	171.9 ± 3.5	<0.001	171.1 ± 3.7	<0.001	199.8 ± 98.6	<0.001	197.0 ± 3.6	0.002	196.5 ± 3.8	0.009
JDDM	145.0 ± 78.2		147.3 ± 3.6		143.5 ± 4.3		171.2 ± 78.2		176.2 ± 5.1		176.5 ± 5.9	
Seaweed (g)												
JDCS	1.9 ± 1.4	0.240	1.8 ± 0.1	0.393	1.8 ± 0.1	0.299	2.2 ± 1.7	0.180	2.3 ± 0.1	0.046	2.3 ± 0.1	0.033
JDDM	1.8 ± 1.4		1.8 ± 0.1		1.7 ± 0.1		2.1 ± 1.6		2.0 ± 0.1		2.0 ± 0.1	
Soybeans/soy products (g)												
JDCS	68.1 ± 48.8	<0.001	67.7 ± 1.8	0.002	66.8 ± 1.9	0.044	74.9 ± 54.3	0.007	75.4 ± 2.0	0.006	75.1 ± 2.1	0.002
JDDM	59.0 ± 43.7		59.3 ± 1.8		60.5 ± 2.2		66.3 ± 42.5		65.4 ± 2.8		62.3 ± 3.2	
Fish (g)												
JDCS	102.4 ± 60.8	<0.001	102.3 ± 2.0	<0.001	101.6 ± 2.2	<0.001	97.2 ± 59.5	<0.001	98.1 ± 2.1	<0.001	97.3 ± 2.2	<0.001
JDDM	72.5 ± 45.3		72.6 ± 2.1		72.7 ± 2.5		68.5 ± 38.7		66.9 ± 2.9		67.5 ± 3.4	
Meat (g)												
JDCS	51.5 ± 37.0	<0.001	51.8 ± 1.7	<0.001	52.2 ± 1.7	<0.001	47.1 ± 39.3	<0.001	46.5 ± 1.6	<0.001	46.1 ± 1.6	<0.001
JDDM	87.4 ± 52.7		87.1 ± 1.8		84.1 ± 2.0		73.8 ± 45.4		74.7 ± 2.2		74.5 ± 2.6	
Eggs (g)												
JDCS	29.8 ± 17.8	0.446	30.1 ± 0.7	0.281	29.8 ± 0.8	0.524	28.0 ± 15.5	0.033	27.6 ± 0.6	0.358	27.7 ± 0.6	0.140
JDDM	29.1 ± 20.9		28.8 ± 0.8		29.0 ± 0.9		25.8 ± 16.7		26.5 ± 0.9		25.8 ± 1.0	
Milk/dairy products (g)												
JDCS	164.7 ± 109.4	<0.001	161.8 ± 4.2	<0.001	160.0 ± 4.2	<0.001	176.7 ± 94.2	<0.001	173.0 ± 3.7	<0.001	173.3 ± 3.7	<0.001
JDDM	125.1 ± 112.5		128.2 ± 4.4		128.1 ± 4.9		132.5 ± 93.7		139.2 ± 5.2		135.9 ± 5.9	
Fruit (g)												
JDCS	120.7 ± 101.0	<0.001	119.0 ± 3.2	<0.001	119.1 ± 3.4	<0.001	147.9 ± 108.1	<0.001	148.7 ± 3.8	<0.001	148.7 ± 3.9	<0.001
JDDM	69.0 ± 66.4		70.8 ± 3.4		70.4 ± 4.0		93.7 ± 70.3		92.2 ± 5.3		92.2 ± 6.2	
Sweets/snacks (g)												
JDCS	15.6 ± 20.3	<0.001	16.3 ± 1.3	<0.001	15.8 ± 1.3	<0.001	20.3 ± 20.5	<0.001	20.4 ± 1.2	<0.001	20.0 ± 1.2	<0.001
JDDM	47.4 ± 42.9		46.6 ± 1.3		50.1 ± 1.5		54.6 ± 42.9		54.4 ± 1.7		56.7 ± 1.9	
Oil (g)												
JDCS	17.0 ± 8.7	<0.001	17.1 ± 0.3	<0.001	17.1 ± 0.3	<0.001	16.7 ± 8.9	<0.001	16.5 ± 0.3	<0.001	16.6 ± 0.3	<0.001
JDDM	12.4 ± 7.6		12.3 ± 0.3		12.2 ± 0.4		12.0 ± 8.0		12.3 ± 0.5		12.1 ± 0.5	

Values are mean ± SD obtained using *t*-test and mean ± SEE obtained using ANCOVA. Model 1 was adjusted for age, HbA1c (%), and BMI (kg/m^2^). Model 2 was adjusted as for Model 1, treated by insulin (yes or no), and treated by OHA (yes or no).

## Data Availability

All data analyzed during this study are included in this paper. Other data are available from the author upon reasonable request.

## Supplementary Materials

The following are available online at https://www.mdpi.com/article/10.3390/nu13103428/s1, Table S1: Clinical characteristics of participants in the Japan Diabetes Complications Study (JDCS, 1996) and the Japan Diabetes Data Management Study (JDDM, 2014-2018) between under 60 and over 60 age, Table S2: Comparison of intake of each nutrient in under 60 and over 60 age participants of JDCS (1996) and JDDM (2014-2018), Table S3: Comparison of intake by major food groups among under 60 and over 60 age participants of JDCS (1996) and JDDM (2014-2018).
