# Supplementary material for: Secular Trends in Dietary Intake over a 20-Year Period in People with Type 2 Diabetes in Japan: A Comparative Study of Two Nationwide Registries; Japan Diabetes Complications Study (JDCS) and Japan Diabetes Clinical Data Management Study (JDDM)
Source: Nutrients. 2021 Sep 28;13(10):3428. doi: 10.3390/nu13103428 (PMC8538089; doi:10.3390/nu13103428)
Supplement: Supplementary file 1 [file nutrients-13-03428-s001.zip › nutrients-1384640-supplementary.pdf]

Supplemental Material

Supplementary Table S1—Clinical characteristics of participants in the Japan Diabetes Complications Study (JDCS, 1996) and the Japan Diabetes Data Management Study (JDDM, 2014–2018) between under 60 and over 60 age.

Men

	Age <60			Age ≧60		
	JDCS (1996) (n =408) Mean ± SD	JDDM (2014-2018) (n =388) Mean ± SD	*P* value	JDCS (1996) (n =396) Mean ± SD	JDDM (2014-2018) (n =363) Mean ± SD	*P* value
Age (y)	52.7 ± 4.7	50.6 ± 5.5	<0.001	64.3 ± 3.0	65.2 ± 3.2	<0.001
Height (cm)	166.6 ± 5.8	170.7 ± 6.2	<0.001	163.8 ± 5.8	167.1 ± 5.8	<0.001
Weight (kg)	63.6 ± 9.0	78.9 ± 15.1	<0.001	60.3 ± 7.9	68.4 ± 10.1	<0.001
BMI (kg/m2)	22.9 ± 2.7	27.0 ± 4.6	<0.001	22.5 ± 2.5	24.5 ± 3.3	<0.001
BMI≧25 kg/m2 (%)	22.5	63.9	<0.001	15.9	42.4	<0.001
Diabetes duration (years)	9.9 ± 6.4	8.8 ± 6.1	0.014	13.1 ± 7.9	12.7 ± 7.4	0.436
HbA1c (%)	8.2 ± 1.4	7.4 ± 1.4	<0.001	8.1 ± 1.1	7.1 ± 1.0	<0.001
Systolic blood pressure (mmHg)	127.9 ± 15.4	126.5 ± 13.9	0.212	134.6 ± 15.3	127.1 ± 15.1	<0.001
Diastolic blood pressure (mmHg)	76.8 ± 10.0	76.2 ± 10.3	0.389	77.5 ± 9.6	70.2 ± 11.6	<0.001
Total serum cholesterol (mg/dl)	195.9 ± 36.9	195.2 ± 35.0	0.800	191.2 ± 32.5	184.3 ± 29.4	0.005
Serum HDL (mg/dl)	52.3 ± 17.4	51.9 ± 14.3	0.720	52.4 ± 15.8	54.7 ± 16.4	0.093
Serum nonHDL (mg/dl)	143.6 ± 38.7	142.9 ± 37.9	0.816	138.8 ± 33.6	129.5 ± 31.1	0.001
Treated by insulin (%)	19.6	21.1	0.072	16.7	18.2	0.064
Treated by OHA without insulin (%)	59.8	54.9	<0.001	60.6	55.1	<0.001
Current smoker (%)	48.3	36.9	<0.001	39.1	20.4	<0.001

Data are mean ± SD.

Women

	Age <60			Age ≧ 60		
	JDCS (1996) (n =344) Mean ± SD	JDDM (2014-2018) (n =159) Mean ± SD	*P* value	JDCS (1996) (n =361) Mean	JDDM (2014-2018) (n =235) Mean	*P* value
Age (y)	53.3 ± 4.7	52.3 ± 5.4	0.040	64.4 ± 2.9	65.4 ± 3.3	<0.001
Height (cm)	153.4 ± 4.9	157.2 ± 5.8	<0.001	152.0 ± 5.1	153.8 ± 4.9	<0.001
Weight (kg)	54.7 ± 8.8	68.5 ± 15.0	<0.001	53.8 ± 7.9	58.4 ± 10.4	<0.001
BMI (kg/m2)	23.2 ± 3.4	27.7 ± 5.8	<0.001	23.3 ± 3.3	24.7 ± 4.4	<0.001
BMI≧25 kg/m2 (%)	28.5	66.0	<0.001	28.0	43.8	<0.001
Diabetes duration (years)	9.1 ± 6.4	8.7 ± 6.5	0.529	11.6 ± 6.9	11.9 ± 7.6	0.592
HbA1c (%)	8.6 ± 1.5	7.4 ± 1.2	<0.001	8.4 ± 1.2	7.2 ± 0.9	<0.001
Systolic blood pressure (mmHg)	130.4 ± 16.5	124.3 ± 16.6	0.001	132.9 ± 16.0	127.4 ± 13.9	<0.001
Diastolic blood pressure (mmHg)	76.4 ± 9.4	72.7 ± 9.8	<0.001	75.7 ± 10.3	69.3 ± 9.1	<0.001
Total serum cholesterol (mg/dl)	211.1 ± 34.3	205.9 ± 34.2	0.158	207.7 ± 32.8	197.5 ± 32.7	0.001
Serum HDL (mg/dl)	58.2 ± 17.3	60.8 ± 16.5	0.175	55.5 ± 16.5	62.3 ± 13.4	<0.001
Serum nonHDL (mg/dl)	152.9 ± 36.9	143.4 ± 34.6	0.020	151.6 ± 31.4	133.9 ± 30.0	<0.001
Treated by insulin (%)	22.1	23.9	0.189	21.9	24.7	0.026
Treated by OHA without insulin (%)	61.9	52.8	<0.001	61.5	48.5	<0.001
Current smoker (%)	8.1	11.3	0.319	8.3	7.2	0.520

Data are mean ± SD.

Supplementary Table S2—Comparison of intake of each nutrient in under 60 and over 60 age participants of JDCS (1996) and JDDM (2014–2018).

Men

		Age <60						Age ≧ 60					
		mean ± SD	*P* value	model 1		model 2		mean ± SD	*P* value	model 1		model 2	
				mean ± SEE	*P* value	mean ± SEE	*P* value			mean ± SEE	*P* value	mean ± SEE	*P* value
Energy (kcal)													
	JDCS	1819 ± 400.6	0.142	1819 ± 23.8	0.226	1812 ± 24.6	0.414	1817 ± 399.6	0.775	1810 ± 22.8	0.522	1808 ± 23.7	0.492
	JDDM	1865 ± 473.8		1864 ± 24.5		1846 ± 28.3		1825 ± 432.6		1833 ± 23.9		1836 ± 28.3	
Carbohydrate													
%Energy	JDCS	58.0 ± 6.5	<0.001	57.9 ± 0.4	<0.001	58.2 ± 0.4	<0.001	58.3 ± 6.2	0.006	58.5 ± 0.3	0.004	58.4 ± 0.4	0.040
	JDDM	55.1 ± 7.4		55.1 ± 0.4		54.9 ± 0.5		57.1 ± 6.2		56.9 ± 0.4		57.1 ± 0.4	
g	JDCS	237 ± 56.7	0.521	237 ± 3.4	0.611	237 ± 3.5	0.366	242 ± 54.1	0.073	241 ± 3.1	0.294	241 ± 3.2	0.537
	JDDM	234 ± 67.3		234 ± 3.5		232 ± 4.0		235 ± 58.9		236 ± 3.3		238 ± 3.9	
Protein													
%Energy	JDCS	15.1 ± 2.3	<0.001	15.0 ± 0.1	0.005	14.9 ± 0.1	0.060	15.3 ± 2.4	0.002	15.4 ± 0.1	0.006	15.4 ± 0.1	0.001
	JDDM	14.4 ± 2.2		14.5 ± 0.1		14.5 ± 0.2		14.8 ± 2.1		14.8 ± 0.1		14.7 ± 0.2	
g	JDCS	68.9 ± 19.9	0.170	68.5 ± 1.1	0.481	67.9 ± 1.1	0.566	70.3 ± 21.6	0.078	70.2 ± 1.1	0.186	70.4 ± 1.2	0.139
	JDDM	67.0 ± 19.4		67.4 ± 1.1		66.8 ± 1.3		67.7 ± 19.0		67.9 ± 1.2		67.4 ± 1.4	
Fat													
%Energy	JDCS	27.0 ± 5.2	<0.001	27.1 ± 0.3	<0.001	26.9 ± 0.3	<0.001	26.3 ± 4.7	<0.001	26.2 ± 0.3	<0.001	26.3 ± 0.3	<0.001
	JDDM	30.6 ± 6.0		30.4 ± 0.3		30.6 ± 0.4		28.1 ± 4.9		28.3 ± 0.3		28.2 ± 0.3	
g	JDCS	54.8 ± 17.1	<0.001	55.3 ± 1.1	<0.001	54.7 ± 1.1	<0.001	53.7 ± 17.1	0.004	53.1 ± 1.0	0.001	53.2 ± 1.0	0.005
	JDDM	63.8 ± 21.9		63.3 ± 1.1		63.1 ± 1.3		57.4 ± 18.1		58.0 ± 1.0		58.0 ± 1.2	
Saturated Fatty Acids (% Energy)													

	JDCS	7.6 ± 1.8	<0.001	7.6 ± 0.1	<0.001	7.6 ± 0.1	<0.001	7.6 ± 1.6	<0.001	7.5 ± 0.1	<0.001	7.6 ± 0.1	<0.001
	JDDM	9.3 ± 2.2		9.3 ± 0.1		9.4 ± 0.1		8.7 ± 2.0		8.7 ± 0.1		8.7 ± 0.1	
Monounsaturated Fatty Acids (% Energy)													
	JDCS	9.0 ± 2.1	<0.001	9.1 ± 0.1	<0.001	9.0 ± 0.1	<0.001	8.5 ± 1.9	<0.001	8.5 ± 0.1	<0.001	8.5 ± 0.1	<0.001
	JDDM	11.1 ± 2.6		11.0 ± 0.1		11.0 ± 0.2		9.8 ± 2.1		9.9 ± 0.1		9.8 ± 0.1	
Polyunsaturated Fatty Acids (% Energy)													
	JDCS	6.5 ± 1.5	<0.001	6.6 ± 0.1	<0.001	6.6 ± 0.1	0.004	6.3 ± 1.4	<0.001	6.3 ± 0.1	<0.001	6.3 ± 0.1	<0.001
	JDDM	6.1 ± 1.3		6.1 ± 0.1		6.2 ± 0.1		5.8 ± 1.2		5.8 ± 0.1		5.8 ± 0.1	
n-6 Fatty Acids (% Energy)													
	JDCS	5.3 ± 1.4	0.006	5.4 ± 0.1	<0.001	5.3 ± 0.1	0.021	5.1 ± 1.3	<0.001	5.1 ± 0.1	<0.001	5.0 ± 0.1	0.001
	JDDM	5.0 ± 1.1		5.0 ± 0.1		5.0 ± 0.1		4.6 ± 1.0		4.6 ± 0.1		4.7 ± 0.1	
n-3 Fatty Acids (% Energy)													
	JDCS	1.5 ± 0.4	<0.001	1.5 ± 0.02	<0.001	1.5 ± 0.02	<0.001	1.5 ± 0.4	<0.001	1.5 ± 0.02	<0.001	1.5 ± 0.02	<0.001
	JDDM	1.1 ± 0.3		1.1 ± 0.02		1.1 ± 0.02		1.2 ± 0.3		1.1 ± 0.02		1.1 ± 0.02	
Fiber(g)													
	JDCS	13.5 ± 5.2	<0.001	13.3 ± 0.2	<0.001	13.1 ± 0.3	0.001	14.7 ± 5.3	<0.001	14.8 ± 0.3	<0.001	14.8 ± 0.3	<0.001
	JDDM	11.5 ± 3.7		11.7 ± 0.3		11.7 ± 0.3		12.9 ± 4.0		12.8 ± 0.3		12.8 ± 0.3	
Salt (g)													
	JDCS	10.1 ± 3.8	<0.001	9.9 ± 0.2	<0.001	9.8 ± 0.2	<0.001	10.9 ± 4.0	<0.001	11.0 ± 0.2	<0.001	11.0 ± 0.2	<0.001
	JDDM	8.0 ± 2.9		8.2 ± 0.2		8.3 ± 0.2		8.8 ± 3.1		8.7 ± 0.2		8.7 ± 0.3	

Values are mean ± SD obtained using t-test and mean ± SEE obtained using ANCOVA.

Model 1 was adjusted for age, HbA1c(%), and BMI(kg/m^2).

Model 2 was adjusted as for model 1 and Treated by insulin (yes or no), Treated by OHA (yes or no).

Women

		Age < 60						Age ≧ 60					
				model 1		model 2				model 1		model 2	
		mean ± SD	*P* value	mean ± SEE	*P* value	mean ± SEE	*P* value	mean ± SD	*P* value	mean ± SEE	*P* value	mean ± SEE	*P* value
Energy (kcal)													
	JDCS	1687 ± 430.8	0.113	1691 ± 24.2	0.283	1682 ± 24.8	0.110	1600 ± 376.5	0.044	1591 ± 20.5	0.020	1591 ± 21.2	0.139
	JDDM	1751 ± 411.7		1743 ± 38.0		1769 ± 43.4		1661 ± 348.9		1674 ± 26.3		1652 ± 31.2	
Carbohydrate													
%Energy	JDCS	54.5 ± 6.2	0.012	54.3 ± 0.4	0.193	54.4 ± 0.4	0.223	55.7 ± 6.1	0.298	55.9 ± 0.3	0.085	55.9 ± 0.3	0.645
	JDDM	53.0 ± 6.1		53.4 ± 0.6		53.5 ± 0.6		55.2 ± 5.5		54.9 ± 0.4		55.6 ± 0.5	
g	JDCS	224 ± 50.8	0.537	223 ± 2.9	0.980	222 ± 2.9	0.559	217 ± 46.1	0.167	217 ± 2.6	0.180	216 ± 2.6	0.247
	JDDM	221 ± 50.5		222 ± 4.5		226 ± 5.1		222 ± 44.3		222 ± 3.3		222 ± 3.9	
Protein													
%Energy	JDCS	16.2 ± 2.4	<0.001	16.2 ± 0.1	<0.001	16.1 ± 0.1	<0.001	16.2 ± 2.4	<0.001	16.2 ± 0.1	<0.001	16.2 ± 0.1	<0.001
	JDDM	14.8 ± 2.2		14.8 ± 0.2		14.8 ± 0.2		15.4 ± 2.1		15.3 ± 0.2		15.1 ± 0.2	
g	JDCS	69.0 ± 24.4	0.055	69.3 ± 1.3	0.046	68.7 ± 1.3	0.225	65.4 ± 20.9	0.347	65.2 ± 1.1	0.608	65.1 ± 1.1	0.258
	JDDM	64.8 ± 17.6		64.2 ± 2.0		65.2 ± 2.3		63.9 ± 16.2		64.2 ± 1.4		62.6 ± 1.6	
Fat													
%Energy	JDCS	29.3 ± 4.7	<0.001	29.5 ± 0.3	<0.001	29.4 ± 0.3	<0.001	28.1 ± 4.8	0.001	27.9 ± 0.3	<0.001	27.9 ± 0.3	0.006
	JDDM	32.2 ± 5.1		31.8 ± 0.4		31.7 ± 0.5		29.4 ± 4.5		29.8 ± 0.3		29.3 ± 0.4	
g	JDCS	55.8 ± 20.0	<0.001	56.3 ± 1.1	0.012	55.9 ± 1.2	0.006	50.8 ± 17.5	0.004	50.0 ± 1.0	<0.001	50.1 ± 1.0	0.020
	JDDM	63.3 ± 20.0		62.0 ± 1.8		62.9 ± 2.0		54.9 ± 16.7		56.1 ± 1.2		54.5 ± 1.5	
Saturated Fatty Acids (% Energy)													
	JDCS	8.5 ± 1.5	<0.001	8.5 ± 0.1	<0.001	8.5 ± 0.1	<0.001	8.2 ± 1.6	<0.001	8.1 ± 0.1	<0.001	8.1 ± 0.1	<0.001

	JDDM	10.0 ± 2.0		9.9 ± 0.2		9.9 ± 0.2		9.0 ± 1.9		9.2 ± 0.1		9.0 ± 0.1	
Monounsaturated Fatty Acids (% Energy)													
	JDCS	9.6 ± 2.0	<0.001	9.7 ± 0.1	<0.001	9.7 ± 0.1	<0.001	9.0 ± 2.0	<0.001	8.9 ± 0.1	<0.001	9.0 ± 0.1	<0.001
	JDDM	11.4 ± 2.3		11.1 ± 0.2		11.1 ± 0.2		10.0 ± 2.0		10.2 ± 0.1		10.0 ± 0.2	
Polyunsaturated Fatty Acids (% Energy)													
	JDCS	7.0 ± 1.5	<0.001	7.1 ± 0.1	<0.001	7.1 ± 0.1	<0.001	6.7 ± 1.5	<0.001	6.7 ± 0.1	<0.001	6.7 ± 0.1	<0.001
	JDDM	6.5 ± 1.3		6.4 ± 0.1		6.3 ± 0.2		6.2 ± 1.2		6.2 ± 0.1		6.1 ± 0.1	
n-6 Fatty Acids (% Energy)													
	JDCS	5.7 ± 1.4	0.004	5.7 ± 0.1	0.002	5.7 ± 0.1	0.001	5.4 ± 1.3	<0.001	5.3 ± 0.1	0.003	5.3 ± 0.1	0.004
	JDDM	5.3 ± 1.1		5.2 ± 0.1		5.2 ± 0.1		5.0 ± 1.0		5.0 ± 0.1		4.9 ± 0.1	
n-3 Fatty Acids (% Energy)													
	JDCS	1.7 ± 0.4	<0.001	1.7 ± 0.02	<0.001	1.7 ± 0.02	<0.001	1.6 ± 0.4	<0.001	1.6 ± 0.02	<0.001	1.6 ± 0.02	<0.001
	JDDM	1.2 ± 0.3		1.1 ± 0.04		1.1 ± 0.04		1.2 ± 0.3		1.2 ± 0.03		1.2 ± 0.03	
Fiber(g)													
	JDCS	15.6 ± 5.4	<0.001	15.5 ± 0.3	<0.001	15.4 ± 0.3	0.002	15.2 ± 5.2	0.007	15.2 ± 0.3	0.038	15.2 ± 0.3	0.020
	JDDM	13.0 ± 4.0		13.2 ± 0.5		13.5 ± 0.5		14.1 ± 3.8		14.2 ± 0.4		13.9 ± 0.4	
Salt (g)													
	JDCS	11.0 ± 4.1	<0.001	11.0 ± 0.2	<0.001	10.9 ± 0.2	<0.001	10.8 ± 3.8	<0.001	10.8 ± 0.2	<0.001	10.8 ± 0.2	<0.001
	JDDM	7.8 ± 2.7		7.8 ± 0.3		8.0 ± 0.4		8.3 ± 2.4		8.3 ± 0.2		8.3 ± 0.3	

Values are mean ± SD obtained using t-test and mean ± SEE obtained using ANCOVA.

Model 1 was adjusted for age, HbA1c(%), and BMI(kg/m^2).

Model 2 was adjusted as for model 1 and Treated by insulin (yes or no), Treated by OHA (yes or no).

Supplementary Table S3—Comparison of intake by major food groups among under 60 and over 60 age participants of JDCS (1996) and JDDM (2014-2018).

Men

	Age < 60						Age ≧ 60					
			model 1		model 2				model 1		model 2	
	mean ± SD	*P* value	mean ± SEE	*P* value	mean ± SEE	*P* value	mean ± SD	*P* value	mean ± SEE	*P* value	mean ± SEE	*P* value
Grains (g)												
JDCS	208.5 ± 58.4	0.030	208.9 ± 3.4	0.045	211.2 ± 3.5	0.001	206.5 ± 57.5	0.001	203.1 ± 3.2	0.156	203.8 ± 3.3	0.090
JDDM	198.8 ± 66.9		198.3 ± 3.5		191.4 ± 4.0		192.3 ± 58.7		196.0 ± 3.3		194.1 ± 4.0	
Potato/aroid (g)												
JDCS	43.9 ± 37.6	<0.001	43.5 ± 1.7	<0.001	42.6 ± 1.7	<0.001	55.6 ± 42.0	<0.001	55.8 ± 2.0	<0.001	55.9 ± 2.1	<0.001
JDDM	20.7 ± 21.5		21.1 ± 1.7		22.0 ± 2.0		25.0 ± 27.1		24.8 ± 2.1		25.7 ± 2.5	
Vegetables total (g)												
JDCS	288.1 ± 161.0	<0.001	282.5 ± 7.5	<0.001	279.5 ± 7.8	<0.001	320.0 ± 179.5	<0.001	321.2 ± 8.3	<0.001	320.5 ± 8.8	<0.001
JDDM	203.0 ± 108.9		209.0 ± 7.7		205.9 ± 9.0		236.1 ± 113.9		234.7 ± 8.8		228.7 ± 10.5	
Green-yellow vegetables (g)												
JDCS	123.2 ± 65.3	<0.001	121.2 ± 3.0	<0.001	119.8 ± 3.1	<0.001	136.5 ± 71.4	<0.001	137.5 ± 3.3	<0.001	136.8 ± 3.5	<0.001
JDDM	65.8 ± 42.5		67.8 ± 3.1		67.3 ± 3.6		82.8 ± 44.4		81.8 ± 3.5		80.7 ± 4.2	
Other vegetables (g)												
JDCS	165.0 ± 96.8	<0.001	161.2 ± 4.8	0.007	159.7 ± 5.0	0.014	183.4 ± 109.1	<0.001	183.8 ± 5.2	<0.001	183.7 ± 5.5	<0.001
JDDM	137.2 ± 79.0		141.2 ± 4.9		138.7 ± 5.8		153.3 ± 76.6		152.9 ± 5.5		148.0± 6.6	
Seaweed (g)												
JDCS	1.8 ± 1.5	0.060	1.8 ± 0.1	0.415	1.7 ± 0.1	0.783	1.9 ± 1.3	0.792	1.9 ± 0.1	0.641	2.0 ± 0.1	0.218
JDDM	1.6 ± 1.3		1.7 ± 0.1		1.7 ± 0.1		1.9 ± 1.5		1.9 ± 0.1		1.8 ± 0.1	

Soybeans/soy products (g)												
	JDCS	65.4 ± 47.6	0.011	64.4 ± 2.5	0.117	62.6 ± 2.6	0.410	70.8 ± 50.0	0.003	71.5 ± 2.6	0.005	71.6 ± 2.7	0.029
	JDDM	57.1 ± 44.8		58.2 ± 2.6		59.0 ± 3.0		61.0 ± 42.4		60.2 ± 2.7		61.4 ± 3.2	
Fish (g)												
	JDCS	101.1 ± 57.5	<0.001	99.5 ± 2.7	<0.001	98.6 ± 2.8	<0.001	103.7 ± 64.1	<0.001	105.0 ± 3.1	<0.001	104.9 ± 3.3	<0.001
	JDDM	63.2 ± 41.0		64.8 ± 2.8		65.2 ± 3.3		82.5 ± 47.5		81.1 ± 3.3		80.7 ± 3.9	
Meat (g)												
	JDCS	54.6 ± 38.2	<0.001	56.3 ± 2.6	<0.001	55.6 ± 2.6	<0.001	48.4 ± 35.5	<0.001	47.1 ± 2.2	<0.001	48.6 ± 2.3	<0.001
	JDDM	99.7 ± 56.5		97.9 ± 2.7		95.1 ± 3.0		74.2 ± 44.8		75.6 ± 2.3		72.3 ± 2.7	
Eggs (g)												
	JDCS	29.4 ± 17.1	0.746	29.7 ± 1.1	0.986	29.1 ± 1.1	0.631	30.2 ± 18.5	0.135	30.3 ± 1.0	0.184	30.4 ± 1.1	0.217
	JDDM	29.9 ± 22.7		29.6 ± 1.1		30.0 ± 1.3		28.2 ± 18.8		28.1 ± 1.1		28.2 ± 1.3	
Milk/dairy product (g)												
	JDCS	159.2 ± 119.3	<0.001	154.8 ± 6.4	0.001	152.1 ± 6.4	0.008	170.4 ± 98.1	<0.001	168.4 ± 5.7	<0.001	167.5 ± 5.6	0.001
	JDDM	118.1 ± 115.9		122.7 ± 6.5		123.2 ± 7.4		132.6 ± 108.5		134.9 ± 6.0		134.4 ± 6.7	
Fruit (g)												
	JDCS	112.6 ± 105.8	<0.001	108.1 ± 4.7	<0.001	107.1 ± 5.0	<0.001	129.0 ± 95.4	<0.001	132.4 ± 4.4	<0.001	133.4 ± 4.6	<0.001
	JDDM	50.5 ± 64.1		55.3 ± 4.9		58.0 ± 5.7		88.9 ± 63.1		85.2 ± 4.7		81.5 ± 5.5	
Sweets/snacks (g)												
	JDCS	14.4 ± 19.2	<0.001	15.3 ± 1.9	<0.001	14.7 ± 2.0	<0.001	16.8 ± 21.4	<0.001	16.8 ± 1.7	<0.001	16.5 ± 1.7	<0.001
	JDDM	50.8 ± 46.7		49.9 ± 2.0		53.2 ± 2.3		43.7 ± 38.2		43.6 ± 1.8		47.5 ± 2.0	
Oil (g)												
	JDCS	17.8 ± 8.9	<0.001	18.1 ± 0.5	<0.001	18.1 ± 0.5	<0.001	16.2 ± 8.3	<0.001	16.0 ± 0.4	<0.001	15.9 ± 0.4	<0.001
	JDDM	13.3 ± 8.0		13.0 ± 0.5		12.7 ± 0.6		11.5 ± 7.1		11.8 ± 0.5		11.9 ± 0.5	

Values are mean ± SD obtained using t-test and mean ± SEE obtained using ANCOVA.

Model 1 was adjusted for age, HbA1c(%), and BMI(kg/m^2).

Model 2 was adjusted as for model 1 and Treated by insulin (yes or no), Treated by OHA (yes or no).

Women

	Age < 60						Age ≧ 60					
	mean ± SD	*P* value	model 1		model 2		mean ± SD	*P* value	model 1		model 2	
			mean ± SEE	*P* value	mean ± SEE	*P* value			mean ± SEE	*P* value	mean ± SEE	*P* value
Grains (g)												
JDCS	175.9 ± 42.3	0.471	174.4 ± 2.4	0.662	174.5 ± 2.4	0.647	170.4 ± 37.5	0.128	169.8 ± 2.2	0.084	169.6 ± 2.2	0.098
JDDM	173.1 ± 40.7		176.4 ± 3.8		176.9 ± 4.2		175.4 ± 39.5		176.3 ± 2.8		176.7 ± 3.3	
Potato/aroid (g)												
JDCS	56.8 ± 43.9	<0.001	57.5 ± 2.2	<0.001	56.2 ± 2.3	<0.001	59.5 ± 55.1	<0.001	58.8 ± 2.6	<0.001	58.8 ± 2.7	<0.001
JDDM	25.7 ± 23.5		24.2 ± 3.5		27.7 ± 4.0		34.9 ± 29.2		36.0 ± 3.4		36.7 ± 4.0	
Vegetables total (g)												
JDCS	358.0 ± 164.9	<0.001	352.1 ± 8.6	<0.001	349.4 ± 8.8	<0.001	337.0 ± 161.1	<0.001	333.4 ± 8.1	<0.001	333.6 ± 8.5	<0.001
JDDM	250.4 ± 117.6		263.1 ± 13.5		268.6 ± 15.4		274.8 ± 115.1		280.3 ± 10.4		276.1 ± 12.5	
Green-yellow vegetables (g)												
JDCS	151.6 ± 66.3	<0.001	149.9 ± 3.5	<0.001	148.7 ± 3.5	<0.001	143.5 ± 64.7	<0.001	142.6 ± 3.3	<0.001	142.4 ± 3.4	<0.001
JDDM	87.7 ± 46.3		91.3 ± 5.4		92.6 ± 6.2		97.9 ± 47.2		99.3 ± 4.2		97.0 ± 5.0	
Other vegetables (g)												
JDCS	206.4 ± 99.6	<0.001	202.2 ± 5.4	0.005	200.7 ± 5.5	0.039	193.5 ± 97.4	0.027	190.8 ± 5.0	0.259	191.2 ± 5.2	0.233
JDDM	162.8 ± 81.9		171.8 ± 8.4		176.0 ± 9.6		176.9 ± 75.2		181.0 ± 6.4		179.1 ± 7.7	

Seaweed (g)													
JDCS	2.2 ± 1.7	0.063	2.2 ± 0.1	0.010	2.2 ± 0.1	0.058	2.3 ± 1.8	0.661	2.3 ± 0.1	0.674	2.3 ± 0.1	0.193
JDDM	1.9 ± 1.5		1.7 ± 0.2		1.8 ± 0.2		2.2 ± 1.7		2.2 ± 0.1		2.1 ± 0.2	
Soybeans/soy products (g)												
JDCS	76.8 ± 53.7	0.007	77.0 ± 2.9	0.027	76.9 ± 3.0	0.029	73.1 ± 54.8	0.160	73.7 ± 2.8	0.138	73.4 ± 2.9	0.047
JDDM	64.5 ± 44.4		64.1 ± 4.6		62.6 ± 5.2		67.5 ± 41.3		66.5 ± 3.6		62.2 ± 4.3	
Fish (g)												
JDCS	99.6 ± 65.3	<0.001	100.7 ± 3.3	<0.001	99.8 ± 3.4	<0.001	94.9 ± 53.5	<0.001	96.4 ± 2.7	<0.001	95.8 ± 2.8	<0.001
JDDM	59.5 ± 35.0		57.0 ± 5.2		59.7 ± 5.9		74.6 ± 40.0		72.3 ± 3.5		71.6 ± 4.1	
Meat (g)												
JDCS	51.8 ± 42.7	<0.001	54.0 ± 2.5	<0.001	52.4 ± 2.6	<0.001	42.6 ± 35.2	<0.001	40.5 ± 2.1	<0.001	40.9 ± 2.1	<0.001
JDDM	88.1 ± 49.2		83.4 ± 4.0		86.3 ± 4.5		64.1 ± 40.0		67.4 ± 2.7		64.2 ± 3.1	
Eggs (g)												
JDCS	27.6 ± 14.8	0.609	27.3 ± 0.9	0.910	27.2 ± 0.9	0.945	28.3 ± 16.3	0.016	27.9 ± 0.9	0.148	28.3 ± 0.9	0.036
JDDM	26.8 ± 17.9		27.5 ± 1.4		27.1 ± 1.6		25.1 ± 15.9		25.7 ± 1.2		24.6 ± 1.3	
Milk/dairy product (g)												
JDCS	178.1 ± 93.0	<0.001	174.0 ± 5.4	0.011	174.1 ± 5.5	0.018	175.4 ± 95.5	<0.001	171.4 ± 5.3	<0.001	171.9 ± 5.2	<0.001
JDDM	137.7 ± 96.6		146.6 ± 8.4		145.6 ± 9.6		128.9 ± 91.8		135.1 ± 6.7		130.3 ± 7.6	
Fruit (g)												
JDCS	143.0 ± 106.6	<0.001	142.8 ± 5.4	<0.001	141.6 ± 5.6	<0.001	152.6 ± 109.5	<0.001	152.4 ± 5.4	<0.001	153.9 ± 5.6	<0.001
JDDM	75.5 ± 62.8		75.9 ± 8.5		81.4 ± 9.7		106.1 ± 72.6		106.2 ± 6.9		103.0 ± 8.3	
Sweets/snacks (g)												
JDCS	22.6 ± 22.3	<0.001	23.1 ± 1.8	<0.001	22.9 ± 1.8	<0.001	18.1 ± 18.4	<0.001	18.4 ± 1.6	<0.001	17.9 ± 1.6	<0.001
JDDM	62.8 ± 46.7		61.7 ± 2.9		64.0 ± 3.2		49.0 ± 39.4		48.6 ± 2.1		50.5 ± 2.3	
Oil (g)												

JDCS	17.8 ± 9.3	<0.001	17.9 ± 0.5	<0.001	17.9 ± 0.5	<0.001	15.6 ± 8.4	<0.001	15.3 ± 0.4	<0.001	15.3 ± 0.5	<0.001
JDDM	13.4 ± 8.8		13.1 ± 0.8		13.0 ± 0.9		11.1 ± 7.2		11.6 ± 0.6		11.4 ± 0.7	

Values are mean ± SD obtained using t-test and mean ± SEE obtained using ANCOVA.

Model 1 was adjusted for age, HbA1c(%), and BMI(kg/m^2).

Model 2 was adjusted as for model 1 and Treated by insulin (yes or no), Treated by OHA (yes or no).
